# Cell proliferation is necessary for the regeneration of oral structures in the anthozoan cnidarian *Nematostella vectensis*

**DOI:** 10.1186/1471-213X-12-34

**Published:** 2012-12-04

**Authors:** Yale J Passamaneck, Mark Q Martindale

**Affiliations:** 1 Kewalo Marine Laboratory, Pacific Biosciences Research Center, University of Hawaii, 41 Ahui Street, Honolulu, HI, 96813, USA

## Abstract

**Background:**

The contribution of cell proliferation to regeneration varies greatly between different metazoan models. Planarians rely on pluripotent neoblasts and amphibian limb regeneration depends upon formation of a proliferative blastema, while regeneration in *Hydra* can occur in the absence of cell proliferation. Recently, the cnidarian *Nematostella vectensis* has shown potential as a model for studies of regeneration because of the ability to conduct comparative studies of patterning during embryonic development, asexual reproduction, and regeneration. The present study investigates the pattern of cell proliferation during the regeneration of oral structures and the role of cell proliferation in this process.

**Results:**

In intact polyps, cell proliferation is observed in both ectodermal and endodermal tissues throughout the entire oral-aboral axis, including in the tentacles and physa. Following bisection, there is initially little change in proliferation at the wound site of the aboral fragment, however, beginning 18 to 24 hours after amputation there is a dramatic increase in cell proliferation at the wound site in the aboral fragment. This elevated level of proliferation is maintained throughout the course or regeneration of oral structures, including the tentacles, the mouth, and the pharynx. Treatments with the cell proliferation inhibitors hydroxyurea and nocodazole demonstrate that cell proliferation is indispensable for the regeneration of oral structures. Although inhibition of regeneration by nocodazole was generally irreversible, secondary amputation reinitiates cell proliferation and regeneration.

**Conclusions:**

The study has found that high levels of cell proliferation characterize the regeneration of oral structures in *Nematostella*, and that this cell proliferation is necessary for the proper progression of regeneration. Thus, while cell proliferation contributes to regeneration of oral structures in both *Nematostella* and *Hydra*, *Nematostella* lacks the ability to undergo the compensatory morphallactic mode of regeneration that characterizes *Hydra*. Our results are consistent with amputation activating a quiescent population of mitotically competent stem cells in spatial proximity to the wound site, which form the regenerated structures.

## Background

Regeneration is mediated by a variety of cell behaviors depending upon the organism. Broadly, the role of cell proliferation in regeneration is generally grouped into two categories: epimorphosis, in which regeneration is mediated by cell proliferation, and morphallaxis, in which regeneration can occur in the absence of cell proliferation. The source of proliferative cells and their differentiation capacity varies among different organisms that display epimorphic regeneration. In planarians, which have become the recent focus of intensive research, regeneration is mediated by neoblasts, pluripotent stem cells which undergo proliferation and subsequent differentiation to replace lost tissues [1]. In the classical system of urodele amphibian regeneration, cell proliferation is also involved, but proliferative cells originate though dedifferentiation of cells [2]. New tissues in the regenerated portion of the limb appear to be derived from the same tissue types in the limb stump, and thus appear to be lineage restricted following an amplification phase [3]. The hydrozoan cnidarian *Hydra*, the best studied exemplar of morphallaxis, can undergo regeneration of oral structures, such as the mouth and tentacles, when cell proliferation is blocked [4,5], although recent work has shown that normal regeneration in *Hydra* is characterized by increased cell proliferation [6,7]. These examples demonstrate the surprisingly large variation in “regenerative strategies” displayed by different metazoan taxa, and beg the question about the evolution of “stable” cell fates and the molecular basis of cell communication and differentiation.

The anthozoan cnidarian *Nematostella vectensis* has recently emerged as a basally divergent metazoan system for studies of developmental patterning [8]. Like *Hydra*, *Nematostella* is a member of the diploblastic clade Cnidaria, which is a sister group to the Bilateria. However, the two species are distantly related, with *Nematostella* being a member of the clade Anthozoa, while *Hydra* is in the clade Hydrozoa. These two clades diverged 600 million years ago, and have likely been evolving independently from one another for longer than have the lineages leading to amphibians and planarians. *Nematostella* has generated interest for the fact that, unlike most other cnidarians, it can be readily spawned in the laboratory, allowing for experimental comparisons of patterning during the developmental events of embryogenesis, asexual reproduction, and regeneration [8-10].

Although a number of functional studies have begun to detail the patterning mechanisms underlying embryogenesis in *Nematostella* (e.g. [11-17]), and the genome has been sequenced [18], the process of regeneration has received comparatively little attention. The present study investigates patterns of cell proliferation during the regeneration of oral structures in *Nematostella*, and evaluates their role in the formation of regenerated structures.

## Results

### Morphogenesis during oral regeneration

**Figure 1:**
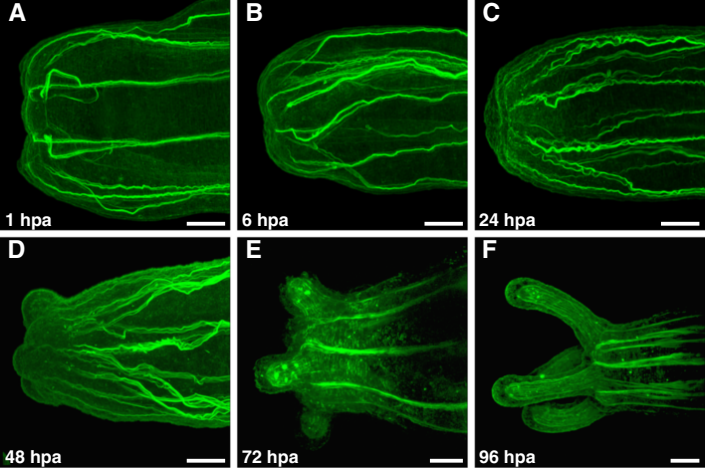
**Morphogenesis of oral regeneration in *Nematostella.*** F-actin stained with BODIPY FL phallacidin (Molecular Probes, Eugene, OR, USA) in all images. (**A**) Cut site on the aboral fragment of bisected polyp, 1 hour post amputation (hpa). Initial epithelial closure has occurred, but severing of the retractor muscles during bisection is evident. (**B**) Cut site 6 hpa. (**C**) Cut site 24 hpa. Relatively little morphological change is evident, although severed ends of retractor muscle have been repaired. (**D**) 48 hpa. Initiation of tentacle buds from outgrowths of the ectoderm and endoderm is evident. (**E**) 72 hpa. Outgrowth of the tentacles continues, including formation of musculature in the tentacular endoderm. Retractor muscles in the body column have reestablished connections with the oral disc. (**F**) 96 hpa. Major repatterning events of regeneration are complete, with reformation of the tentacles, mouth, and pharynx. Scale bars = 50 *μ*m.

Wound closure is initiated rapidly after bisection of polyps, with the edges of the wound site coming together almost immediately after bisection (Figure 1A). Shortly after bisection, oriented fibers of the myoepithelial cells present at the wound site end abruptly and unevenly due to severing during amputation (Figure 1B). During the initial 24 to 36 hours following amputation, relatively few overt changes in morphology are observed, except that the retractor muscle fibers recede from the wound site (Figure 1C). Within 36 to 48 hours after bisection, tentacle buds form as outpocketings of ectoderm and endoderm around the edges of the wound site (Figure 1D). This generally consisted of four tentacle buds, distributed evenly around the reforming oral end of the polyp. During this period the reforming pharynx also first becomes distinguishable as a solid mass of cells underlying the future oral opening. By 72 hours post amputation the tentacles have continued their outgrowth, longitudinal muscles have begun to form in the tentacle endoderm, and the bundled retractor muscles in the body column endoderm have reestablished connections with the oral disc (Figure 1E). By 96 hours post amputation, repatterning of the oral structures is essentially complete (Figure 1F). The tentacles, mouth, pharynx, and musculature have all been reestablished, and the polyps are able to feed. The tentacles may continue to grow for a few days after this point.

### Cell proliferation in intact polyps

**Figure 2:**
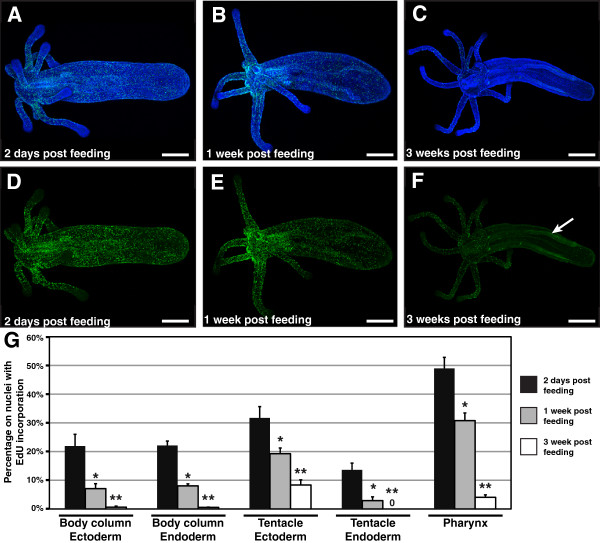
**Cell proliferation in intact polyps**. (**A**-**C**) Nuclei of proliferating cells (green) labeled with the thymidine analog EdU (Molecular Probes, Eugene, OR, USA), and all nuclei counterstained with Hoechst (blue). (**D**-**F**) Nuclei of proliferating cells labeled with EdU (green). (**A**, **D**) Polyp two days after feeding (2 dpf) with *Artemia* nauplii. Widespread proliferation is visible throughout the polyp, including in the tentacles and the physa. Proliferating cells are absent only from the distal tips of the tentacles (**B**, **E**) One week after the last feeding (1 wpf), proliferating cells are still detected throughout the polyp. The numbers of proliferating cells are particularly high in the pharynx and in the oral disc where tentacle buds are emerging. (**C**, **F**) Three weeks after the last feeding (3 wpf). Proliferating cells are detected in the tentacles, but are nearly absent from the remainder of the oral disc, pharynx and body column. Weak signal in the directive mesenteries (arrow) is attributable to autofluorescence, detected due to increased gain used during laser scanning confocal microscopy of the sample. (**G**) Chart of tissue specific levels of cell proliferation in intact polyps 2 dpf, 1 wpf, and 3 wpf. Values are the mean of measurements for 3 polyps at each time point. Error bars: standard error. One asterisk: *p* value < 0.05; Student’s *t*-test for 2 dpf versus 1 wpf. Two asterisks: *p* value < 0.05; Student’s *t*-test for 1 wpf versus 3 wpf. Scale bars = 200 *μ*m.

Cell proliferation was detected by labeling animals with the thymidine analog 5-ethynyl-2^′^-deoxyuridine (EdU), which is incorporated into genomic DNA during S-phase [19]. Polyps labeled with EdU for 30 minutes at two days after feeding with *Artemia* nauplii display extensive EdU incorporation in cells throughout the body (Figure 2A, D). In the body column, labeled cells show a fairly even distribution from below the oral ring to the tip of the physa (Figure 2D). Concentrations of labeling are highest in the pharynx, with 49.0% ± 3.9% of cells having EdU incorporation (Figure 2D, G). High concentrations of labeling are also observed in the tentacle ectoderm (Figure 2D, G). In the tentacles, labeling is greater in the ectoderm (31.7% ± 3.9%) than in the endoderm (13.6% ± 2.3%), while in the body column levels of labeling in both tissue layers are nearly the same (ectoderm: 21.9% ± 4.1%; endoderm: 22.1% ± 1.5%; Figure 2G). The one region of the body where EdU labeling appears to be absent is at the tips of the tentacles (Figure 2D).

Animals labeled one week after feeding continue to display cell proliferation though the body (Figure 2B, E), although at lower levels than at two days after amputation (Figure 2G). High concentrations of proliferating cells are observed in the oral ring where new tentacles are forming, and at the base of existing elongate tentacles (Figure 2E). Again, proliferating cells are almost completely absent from the distal tips of elongate tentacles (Figure 2E). For all tissues the percentage of EdU labeled cells was significantly lower at one week after feeding than at two days after feeding (*p* value < 0.05; Figure 2G).

By three weeks after feeding, the percentage of proliferating cells in polyps has decreased dramatically (Figure 2C, F, G). EdU labeled cells are nearly absent from the body column ectoderm and endoderm, including the mesenteries (Figure 2F, G). More cell proliferation is observed in the tentacle ectoderm and pharynx, but here also the proportion of proliferating cells is decreased from one week after feeding (Figure 2F, G). For all tissues the percentage of EdU labeled cells was significantly lower at three weeks after feeding than at one week after feeding (*p* value < 0.05; Figure 2G)

### Cell proliferation during oral regeneration

**Figure 3:**
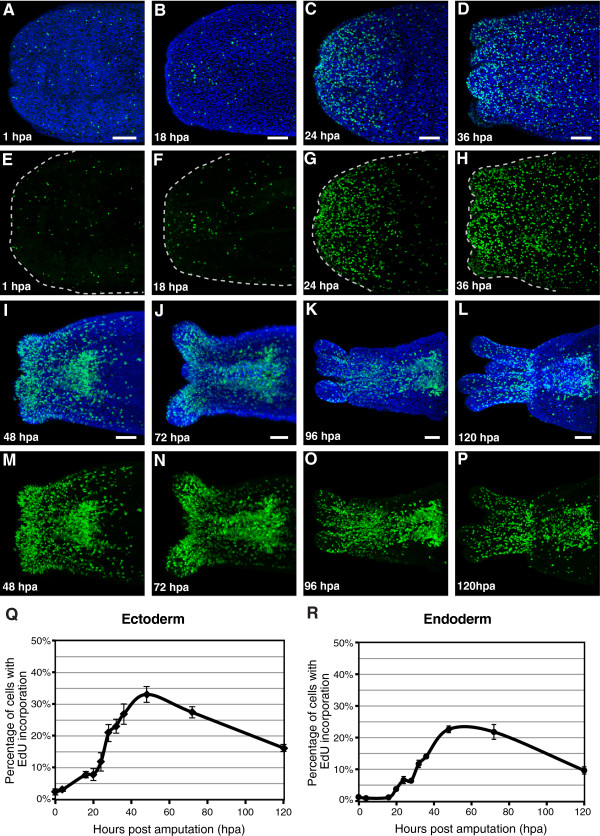
**Cell proliferation in regenerating polyps.** (**A**-**D**; **I**-**L**) Nuclei of proliferating cells (green) labeled with the thymidine analog EdU (Molecular Probes, Eugene, OR, USA), and all nuclei counterstained with Hoechst (blue). (**E**-**H**; **M**-**P**) Nuclei of proliferating cells labeled with EdU (green). (**A**, **E**) Cut site on the aboral fragment of bisected polyp, 1 hpa. Low numbers of proliferating cells are observed. (**B**, **F**) Cut site 18 hpa, with low numbers of proliferating cells. (**C**, **G**) 24 hpa, high numbers of proliferating cells are detectable close to the site of amputation. (**D**, **H**) 36 hpa, high numbers of proliferating cells are detectable in the region of the cut site, including in the emerging tentacle buds. (**I**, **M**) 48 hpa, high numbers of proliferating cells are detectable in the regenerating oral structures, including the tentacle buds and pharynx. (**J**, **N**) 72 hours after amputation. (**K**, **O**) 96 hpa. (**L**, **P**) 120 hpa, fewer proliferating cells are detected. (**Q**) Chart of levels of cell proliferation in the ectoderm during regeneration. (**R**) Chart of levels of cell proliferation in the endoderm during regeneration. Values are the mean of measurements for at least 4 polyps at each time point. Error bars: standard error. Scale bars = 50 *μ*m.

Measurements of cell proliferation during regeneration of oral structures were made with animals bisected through the polyp body column three weeks after their final feeding with *Artemia* nauplii, at which point cell proliferation was nearly absent from the region of the cut. There is slow increase in the percentage of proliferating cells in the ectoderm near the wound site during the first 18 to 20 hours immediately following amputation, while there is no increase in the level of proliferation in the endoderm during the first 18 hours after amputation (Figure 3A, B, E, F, Q, R).

By 24 hours after amputation there is a marked increase in the percentage of proliferative cells in both the ectoderm and endoderm close to the wound site (Figure 3C, G, Q, R). The percentage of proliferating cells in both the ectoderm and endoderm continues to increase rapidly between 24 hours and 48 hours post amputation (Figure 3Q, R), reaching a maximum at 48 hours after amputation in the ectoderm (Figure 3Q), and between 48 hours and 72 hours post amputation in the endoderm (Figure 3R). Between 24 hours and 36 hours post amputation, proliferative cells are distributed fairly evenly in the tissue near the wound site (Figure 3C, G, D, H). By 48 hours post amputation the majority of proliferating cells in the ectoderm are located in the tentacle buds, while the majority of proliferating cells in the endoderm are located in the reforming pharynx (Figure 3I, M). This distribution of proliferating cells is maintained through 120 hours post amputation (Figure 3J-L, N-P), when regeneration of oral structures is nearly complete and the percentage of proliferating cells in both the endoderm and ectoderm has decreased from its maximum (Figure 3Q, R).

### Cell proliferation is required for *Nematostella* regeneration

**Figure 4:**
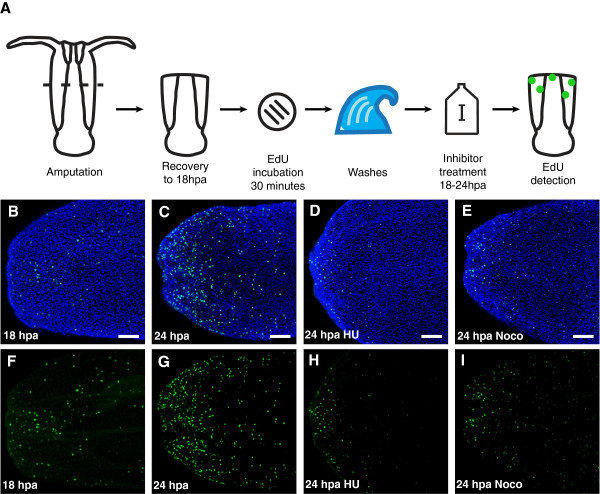
**Efficacy of hydroxyurea and nocodazole in blocking cell proliferation.** (**A**) Schematic of experiments. (**B**-**E**) Nuclei of proliferating cells (green) labeled with the thymidine analog EdU, and all nuclei counterstained with Hoechst (blue). (**F**-**I**) Nuclei of proliferating cells labeled with EdU (green). (**B**, **F**) Region of the wound site in a polyp incubated with EdU for 30 minutes at 18 hpa, and fixed immediately after EdU incubation for detection. (**C**, **G**) Region of the wound site in a polyp incubated with EdU for 30 minutes at 18 hpa, washed and maintained in 1/3x filtered seawater until 24 hpa, and fixed 24 hpa. (**D**, **H**) Region of the wound site in a polyp incubated with EdU for 30 minutes at 18 hpa, washed and maintained in 1/3x filtered seawater with 20 mM hydroxyurea until 24 hpa, and fixed 24 hpa. (**E**, **I**) Region of the wound site in a polyp incubated with EdU for 30 minutes at 18 hpa, washed and maintained in 1/3x filtered seawater with 0.1 *μ*M nocodazole until 24 hpa, and fixed 24 hpa. Scale bars = 50 *μ*m.

To evaluate the role of cell proliferation in regeneration, treatments were performed with two inhibitors of cell proliferation, hydroxyurea and nocodazole. Efficacy of these two compounds in blocking proliferation was initially established by pulse-chase experiments with EdU labeling and inhibitor treatment (Figure 4A). Polyps were bisected three weeks after their final feeding, and incubated with EdU for 30 minutes at 18 hours after amputation. Following incubation, unincorporated EdU was washed out with several exchanges of 1/3x filtered seawater and polyps were maintained in 1/3x filtered seawater with or without inhibitor for 6 hours, until 24 hours post amputation, when they were fixed and visualized. Control polyps maintained in 1/3x seawater for 6 hours after incubation with EdU showed a significant increase in the percentage of labeled nuclei, as compared with polyps fixed immediately after incubation with EdU at 18 hours post amputation (3.3% ± 0.1% at 18 hpa versus 11.7% ± 0.5% at 24 hpa; *p* value = 3.1 x 10^-5^) (Figure 4B, C, F, G). In contrast, polyps maintained in 20 mM hydroxyurea or 0.1 μM nocodazole showed a significantly lower percentage of labeled nuclei at 24 hours post amputation, as compared with control polyps at 24 hours post amputation (hydroxyurea: 5.2% ± 0.4%, *p* value = 2.8 x 10^-5^; nocodazole: 4.5% ± 0.7%, *p* value = 9.0 x 10^-5^; Figure 4D, E, H, I). Incubation with EdU at the end of incubation with hydroxyurea or nocodazole resulted in no detectable incorporation (data not shown). These data show that both treatments effectively prevent cell proliferation, as measured by proliferation of labeled cells during treatment and by the incorporation of EdU during S-phase.

**Figure 5:**
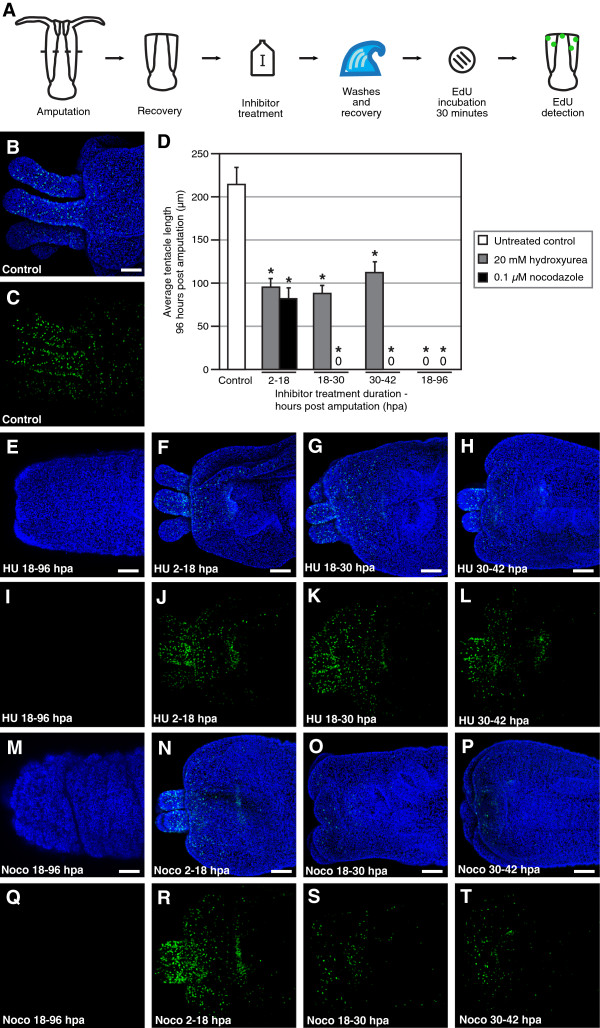
**Cell proliferation is required for regeneration of oral structures.** (**A**) Schematic of experiments. (**B**; **E**-**H**; **M**-**P**) Nuclei of proliferating cells (green) labeled with the thymidine analog EdU at 96 hpa, and all nuclei counterstained with Hoechst (blue). (**C**; **I**-**L**; **Q**-**T**) Nuclei of proliferating cells labeled with EdU at 96 hpa (green). (**B**, **C**) Regeneration of oral structures in untreated control polyps 96 hpa. (**D**) Chart of average tentacle lengths in control and inhibitor treated polyps 96 hpa. Values are the mean of measurements for at least 3 polyps at each time point. Error bars: standard error. Asterisk: *p* value < 0.05; Student’s *t*-test of treatment versus control. (**E**, **I**) Failure to undergo regeneration of oral structures after treatment with 20 mM hydroxyurea from 18 hpa to 96 hpa. (**F**, **J**) Regeneration of oral structures after treatment with 20 mM hydroxyurea from 2 hpa to 18 hpa. (**G**, **K**) Regeneration of oral structures after treatment with 20 mM hydroxyurea from 18 hpa to 30 hpa. (**H**, **L**) Regeneration of oral structures after treatment with 20 mM hydroxyurea from 30 hpa to 42 hpa. (**M**, **Q**) Failure to undergo regeneration of oral structures after treatment with 0.1 *μ*M nocodazole from 18 hpa to 96 hpa. (**N**, **R**) Regeneration of oral structures after treatment with 0.1 *μ*M nocodazole from 2 hpa to 18 hpa. (**O**, **S**) Failure to undergo regeneration of oral structures after treatment with 0.1 *μ*M nocodazole from 18 hpa to 30 hpa. (**P**, **T**) Failure to undergo regeneration of oral structures after treatment with 0.1 *μ*M nocodazole from 30 hpa to 42 hpa. Scale bars = 50 *μ*m.

Inhibition of cell proliferation with hydroxyurea and nocodazole was initially conducted with continuous treatment from 18 hours after amputation to 96 hours after amputation. 96 hours after amputation, polyps were fixed and tentacle length was measured. In untreated, control polyps, the average tentacle length 96 hours after amputation was 214 μm ± 34 μm (Figure 5B-D). Continuous treatment with 20 μM hydroxyurea resulted in no detectable outgrowth of tentacles at 96 hours after amputation (Figure 5D, E, I). There was no detectable necrosis, suggesting that the effect was due specifically to inhibition of cell proliferation, and not more generally toxicity. Likewise, treatment with 0.1 μM nocodazole completely blocked reformation of the tentacles (Figure 5D, M, Q). In the case of the nocodazole treatment, there was no evidence of widespread necrosis, such as sloughing of the ectoderm, but there was folding of the ectoderm and an overall decrease in polyp size (data not shown), suggesting that an intact microtubule system is needed to retain normal morphological integrity.

To test the importance of cell proliferation during different periods of the regeneration process, amputated polyps were exposed to pulse treatments of hydroxyurea or nocodazole, washed with 1/3x filtered seawater to remove the inhibitor, and incubated until 96 hours after amputation to determine effects on tentacle growth (Figure 5A). Polyps were also incubated with EdU at 96 hours after amputation to test for the perdurance of effects on cell proliferation after pulse treatments with inhibitors and the re-initiation of cell proliferation. Pulse treatments were performed over three periods: from 2 to 18 hours after amputation, when levels of cell proliferation are similar to those before amputation; from 18 to 30 hours after amputation, when there is a dramatic increase in the levels of proliferating cells; and from 30 to 42 hours after amputation, when the tentacle buds begin to form.

Treatment with hydroxyurea during all three periods produced similar results, with no statistical difference in tentacle lengths between treatments (*p* value = 0.30, one-way ANOVA; Figure 5D, F-H). In each case the tentacle length at 96 hours after amputation was approximately half that of untreated polyps the same time after amputation (control: 214.3 μm ± 19.7 μm; hydroxyurea 2–18 hpa: 95.3 μm ± 8.7 μm; hydroxyurea 18–30 hpa: 88.0 μm ± 9.5 μm; hydroxyurea 30–42 hpa: 112.2 μm ± 12.2 μm). All three treatments also showed similar levels of cell proliferation, primarily in the tentacles, oral disc, and pharynx (Figure 5J-L). In contrast, in nocodazole treatments tentacle regeneration was only observed in treatments from 2 to 18 hours after amputation (81.9 μm ± 12.0 μm; Figure 5D, N). In both later treatments, from 18 to 30 hours after amputation and 30 to 42 hours after amputation, no tentacle growth was observed by 96 hours (Figure 5D, O, P).

In polyps treated with nocodazole from 2 to 18 hours after amputation, the number of proliferating cells at 96 hours after amputation was comparable to that in all three pulse treatments with hydroxyurea (Figure 5J, K, L, R). In the later treatments, from 18 to 30 hours and 30 to 42 hours, proliferating cells were observed at 96 hours, but the number was greatly reduced (Figure 5S, T). No regeneration of oral structures was observed in these later nocodazole treated polyps after several weeks in 1/3x seawater (data not shown).

### Regeneration can be reinitiated in drug treated animals following secondary amputation

**Figure 6:**
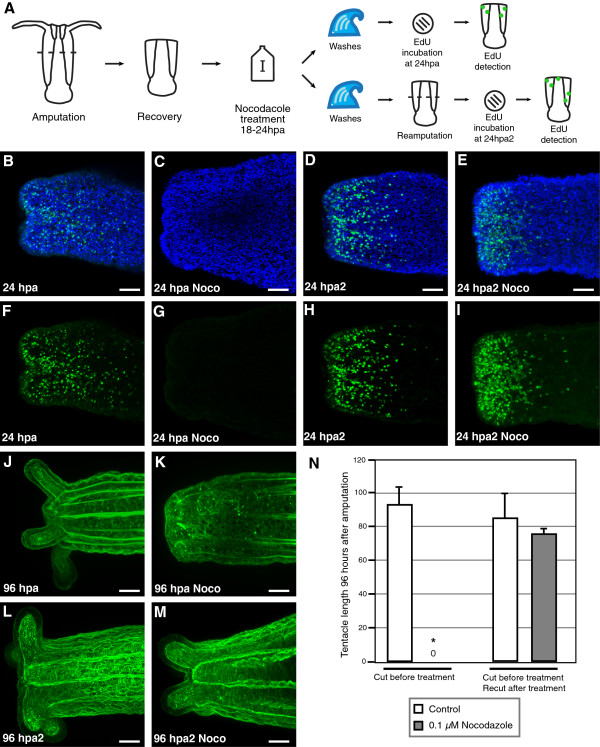
**Re-initiation of regeneration following secondary bisection.** (**A**) Schematic of experiments. (**B**-**E**) Nuclei of proliferating cells (green) labeled with the thymidine analog EdU, and all nuclei counterstained with Hoechst (blue). (**F**-**I**) Nuclei of proliferating cells labeled with EdU (green). In all experiments, polyps were incubated with EdU for 30 minutes immediately prior to fixation. (**B**, **F**) Region of the wound site in a control polyp maintained in 1/3 seawater until 24 hpa. (**C**, **G**) Polyp incubated with 0.1 *μ*M nocodazole from 18 hpa to 24 hpa and fixed. (**D**, **H**) Polyp maintained in 1/3x seawater until 24 hpa, re-bisected, and maintained in 1/3x seawater until 24 hours after secondary amputation (hpa2). (**E**, **I**) Polyp incubated with 0.1 *μ*M nocodazole from 18 hpa to 24 hpa, re-bisected, and maintained in 1/3x seawater until 24 hpa2. (**J**-**M**) Staining of F-actin stained with BODIPY FL phallacidin. (**J**) Regeneration of oral structures in a control polyp maintained in 1/3 seawater until 96 hpa. (**K**) Polyp incubated with 0.1 *μ*M nocodazole from 18 hpa to 24 hpa, washed, and maintained until 96 hpa. (**L**) Polyp maintained in 1/3x seawater until 24 hpa, re-bisected, and maintained in 1/3x seawater until 96 hpa2. (**M**) Polyp incubated with 0.1 *μ*M nocodazole from 18 hpa to 24 hpa, re-bisected, and maintained in 1/3x seawater until 96 hpa2. (**N**) Chart of average tentacle lengths 96 hours after initial or secondary amputation. Values are the mean of measurements for at least 4 polyps at each time point. Error bars: standard error. Asterisk: *p* value < 0.05; Student’s *t*-test of treatment versus control. Scale bars = 50 *μ*m.

Secondary amputations were performed on nocodazole treated polyps, which do not normally undergo regeneration, to test the ability of a new wound healing event to reinitiate cell proliferation and regeneration (Figure 6A). 24 hours after the initial bisections, aboral fragments were bisected again. This procedure was performed both on polyps that had been maintained in 1/3x seawater, as well as those that had been incubated in nocodazole from 18 to 24 hours after the initial amputation.

Polyps incubated with EdU at 24 hours after amputation showed levels of cell proliferation comparable with those observed in other experiments, described above (Figure 6B, F). However, when polyps were incubated with EdU following nocodazole treatment from 18 to 24 hours after amputation, no detectable cell proliferation was observed (Figure 6C, G). 96 hours after amputation, untreated polyps displayed regeneration of the tentacles (91.5 μm ± 11.8 μm), while nocodazole treated polyps showed no outgrowth of tentacles (Figure 6J, K, N).

Untreated polyps which were bisected a second time showed high levels of cell proliferation at the wound site 24 hours after the second amputation (Figure 6D, H). Polyps bisected a second time following incubation with nocodazole showed comparable levels of cell proliferation 24 hours after the second bisection (Figure 6E, I). Both control and nocodazole treated polyps underwent regeneration after the second amputation, with similar tentacle lengths achieved 96 hours after the second amputation (recut control: 84.0 μm ± 15.0 μm; recut nocodazole treated: 74.8 μm ± 0.5 μm; *p* value = 0.61; Figure 6L-N).

## Discussion

*Nematostella* polyps demonstrate the ability to undergo rapid regeneration of oral structures, including the tentacles, mouth, and pharynx after amputation. Initial wound closure is very rapid, beginning almost immediately after bisection of the animal. The finding that muscle fibers recede from the wound site shortly after amputation is consistent with observations of regeneration made in *Myosin Heavy Chain1* transgenic polyps by Renfer et al. [20]. Following initial wound closure, relatively little morphological change is seen until the tentacle buds begin to emerge, two to three days later. Following this, the tentacles grow progressively and the mouth and pharynx are reformed.

### Cell proliferation in intact polyps

Cell proliferation occurs throughout the polyp, including in the mouth, oral disc, tentacles and physa. The one part of the polyp which appears to be relatively free of proliferating cell is the tentacle tips, which are densely packed with fully differentiated cnidocytes [21]. These results suggest that growth occurs throughout the polyp, rather than at a localized growth zone. This distribution of proliferating cells throughout the polyp is comparable to previous results from another anemone, *Aiptasia*, where tritiated thymidine uptake was observed along the length of the oral-aboral axis, including in the tentacles [22]. As in *Nematostella*, cell proliferation in *Aiptasia* was found to be highest in the tentacles and the oral disc. Singer [22] reported relatively little labeling with tritiated thymidine in the endoderm of *Aiptasia* as compared with the ectoderm. Whether this represents a distinct difference in rates of proliferation in the two tissue layers, or is due to a technical limitation of the experiments, is uncertain. Although levels of EdU incorporation in *Nematostella* were greater in the ectoderm than in the endoderm of the tentacles, levels of incorporation were nearly equivalent in the body column ectoderm and endoderm (including the mesenteries). Cell proliferation in tentacles has also been observed in the scyphozoan *Aurelia* during metamorphosis of the planula into the primary polyp [23]. This is in contrast with studies of *Hydra*, where cell proliferation is localized to the body column, and is notably absent from the tentacles, as well as the pedal disc. Likewise, an absence of proliferating cells in the tentacles has also been observed in other hydrozoans, including in the polyps of *Hydractinia*[24], *Tubularia*[25], and *Podocoryne*[26], and in the hydromedusae of *Clytia*[27] and *Podcoryne*[26].

Localized cell proliferation in the body column, and absence from the tentacles, appears to be a common feature within hydrozoans, and may be the ancestral state for the Hydrozoa. Our finding of cell proliferation within the tentacles and physa of *Nematostella*, along with previous work in *Aiptasia*[22], demonstrates that growth in actinarian anthozoans occurs locally throughout the body, rather than through the movement of cells from the body column to the extremities, as is the case in hydrozoans. Additional taxonomic sampling will be necessary to determine the ancestral state for polyp growth in the Cnidaria in general.

### Cell proliferation during regeneration of oral structures

We have found that in *Nematostella* polyps there is a slow increase in the percentage of proliferating ectodermal cells during the initial 20 hours following amputation of the oral structures. During the period from 20 to 24 hours after amputation there is a rapid increase in the percentage of proliferating cells in the ectoderm close to the wound site. This increase in the percentage of proliferating cells reaches a maximum around 48 hours after amputation, and slowly decreases thereafter, as the tentacles, mouth, and pharynx are reformed. In the endoderm, cell proliferation remains at a low level until 16 hours after amputation, and then undergoes a steady increase until at least 48 hours after amputation, before gradually decreasing during the latter morphogenetic period of regeneration. Therefore, the greatest increase in the levels of cell proliferation occur in the period after wound healing and before or during the early stages of oral structure morphogenesis. In *Aiptasia*, levels of proliferation during regeneration were likewise reported to increase dramatically after the initial period of wound healing [22]. Interestingly, in *Aiptasia* proliferation was reported to be localized primarily to the ectoderm, with only minimal proliferation observed in the endoderm [22]. In *Nematostella,* the overall trend in levels of cell proliferation during regeneration is comparable between the ectoderm and endoderm, although a higher maximum level of proliferation is reached in the ectoderm.

### Cell proliferation is required for regeneration of oral structures

Pulse-chase experiments with EdU labeling and inhibitor treatment demonstrated that both hydroxyurea and nocodazole were effective in inhibiting cell proliferation, and both compounds were utilized to evaluate the role of cell proliferation during regeneration of the oral structures. Extended incubations from 18 to 96 hours after amputation with either hydroxyurea or nocodazole resulted in no regeneration of the tentacles, mouth, or pharynx, evidencing the requirement of cell proliferation for regeneration of each of these structures. Pulse-chase experiments with inhibitors demonstrated that cell proliferation through the initial 42 hours after amputation appears to contribute to regeneration. With hydroxyurea, comparable reductions in tentacle length at 96 hours after amputation were observed following treatments from 2 to 18 hours after amputation, the period of wound healing, from 18 to 30 hours after amputation, the period of increased proliferation, and from 30 to 42 hours after amputation, the period of initial tentacle bud formation. In each case, the effects of hydroxyurea were reversible, with cell proliferation and regeneration proceeding after removal of the inhibitor. While nocodazole treatment from 2 to 18 hours after amputation likewise reduced tentacle length, treatments from 18 to 30 hours after amputation or from 30 to 42 hours after amputation permanently blocked the progression of regeneration. These results suggest that cell proliferation during the initial wound healing stage contributes to, but is not required for, the progression of regeneration. However, the increased levels of cell proliferation which are observed later are essential for the progression of regeneration. It is uncertain whether the differences in the effects of these two inhibitors may be attributable to their mechanisms of action. Hydroxyurea, which blocks DNA synthesis, has been shown to be reversible in cell culture following removal of the inhibitor [28,29]. Nocodazole, which blocks mitosis by binding tubulin and preventing microtubule polymerization, has been shown to be reversible in vitro and in cell cultures [30,31]. However, our results suggest that it permanently blocks proliferation in *Nematostella* under the conditions used. The relative absence of EdU incorporation following nocodazole treatment suggests that cells at the wound site may undergo multiple rounds of proliferation, which are blocked by exposure to the inhibitor. Experiments conducted during the study to address this question were equivocal (data not shown). Future investigations will be required to determine the precise fate of proliferating cells over the course of regeneration.

We were able to overcome the inhibition of regeneration by nocodazole though secondary amputation. Polyps that were re-amputated following exposure to nocodazole initiated cell proliferation at the new wound site and grew tentacles at rates comparable to polyps in control treatments. These results suggest that initiation of cell proliferation and regeneration may be a localized event linked to wound closure and healing. It appears likely that a distinct population of cells at the secondary wound site were unaffected by the nocodazole treatment and begin to proliferate once stimulated by the secondary amputation. The nature of these cells, whether they are stem cells or dedifferentiated cells that have reentered the cell cycle, remains to be determined. One factor that may be mediating the response to amputation is the canonical Wnt/ß-catenin pathway, as treatments with alsterpaullone, which prevents degradation of cytosolic ß-catenin, have been shown to bias *Nematostella* regeneration towards oral fates [32].

The role of cell proliferation in *Nematostella* appears to be distinct from that in the hydrozoan cnidarian *Hydra*. Regeneration in *Hydra* has classically been described as morphallactic, based upon numerous studies have demonstrated the ability of *Hydra* to undergo reformation of oral structures in the absence of cell proliferation [4,5,33-36]. However, in unperturbed animals increased cell proliferation at the wound site has been shown to characterize regeneration response both shortly after amputation in response to Wnt3 signaling from apoptotic cells [7,37], and in association with neurogenesis 24 to 48 after amputation, prior to the emergence of the tentacle buds [6,38]. Although regeneration of oral structures can proceed in *Hydra* in the absence of cell proliferation, a number of abnormalities, including changes in the number of tentacles formed, have been observed in polyps where cell proliferation has been inhibited to produce polyps lacking nerve cells [39]. In *Nematostella* we have found an absence of any morphallactic response, with cell proliferation being absolutely required for the regeneration of oral structures, including the tentacles, mouth, and pharynx, in the experiments performed. A role for cell proliferation in regeneration has also been suggested in the scyphozoan *Aurelia*, where hydroxyurea treatment inhibits the reformation of polyps from isolated tentacle fragments [40]. These results suggest that cell proliferation may be a common feature of oral regeneration in the Cnidaria in general, with *Hydra* having acquired the ability to undergo regeneration through a compensatory morphallactic mode.

## Conclusions

The results of this study demonstrate that increased cell proliferation occurs during the regeneration of oral structures in *Nematostella*, and is necessary for regeneration to proceed. This epimorphic mode of regeneration differs from the morphallactic regeneration potential in *Hydra*, which can proceed without the contribution of cell proliferation. Future studies will be needed to determine the origin and character of the proliferative cells that participate in *Nematostella* regeneration.

## Methods

### Animal care

Regeneration experiments were performed with polyps raised from in vitro fertilized embryos, and fed weekly with freshly hatched *Artemia* nauplii until they were approximately 5-10 mm in length when relaxed and had eight or ten tentacles. Amputation was performed three weeks after the last feeding by relaxing the polyps with MgCl_2_ for ten minutes, and bisecting them a scalpel. In all cases the cut was made orthogonally to the oral-aboral axis of the polyp, at a point approximately 0.5 mm aboral of the pharynx. Amputated aboral fragments less than 2.5 mm in length were excluded from experiments as they failed to produce a robust regeneration response. Amputated fragments were washed several times with 1/3x filtered seawater, and maintained at 22°C for all experiments.

### Detection of cell proliferation

Proliferating cells were labeled by incubation with the thymidine analog 5-ethynyl-2^′^-deoxyuridine (EdU), which was subsequently labeled fluorescently with the Click-It EdU Imaging Kit (Invitrogen, Carlsbad, CA, USA) [19]. EdU incubations for immediate fixation were performed with 100 μM of EdU in 1/3 seawater for 30 minutes. Following incubation, polyps were relaxed in MgCl_2_ for ten minutes prior to fixation. For pulse-chase experiments, polyps were incubated with 10 μM EdU in 1/3x seawater for 30 minutes, washed 3 times with 1/3x seawater, and maintained in 1/3x seawater with 100 μM thymidine until fixation. Fixations were performed with ice-cold 4% paraformaldehyde and 0.2% glutaraldehyde in 1x PBS for two minutes, followed by 4% paraformaldehyde in 1x PBS for one hour. Detection of EdU with Alexa azide fluorophores was performed following the manufacturer’s protocols. Following detection, polyps were washed three times in 1x PBS with 0.1% Triton X-100 (PTx), and subsequently all nuclei were counterstained with Hoechst 33342 (Invitrogen, Carlsbad, CA, USA) at 10 μg/ml in PTx for 2 hours. Polyps were cleared for imaging with a series of glycerol in 1xPBS at concentrations of 20%, 40%, 60% and 80%. Confocal imaging was performed using a Zeiss LSM 710 microscope (Carl Zeiss Microimaging, Inc., Thornwood, NY, USA) with either a 20x/0.8 NA dry objective or a 40×/1.3 NA oil immersion objective.

### Measurement of cell proliferation

To determine the percentage of EdU labeled cells in regenerating polyps, counts were performed in the area extending 350 μm from the initial amputation site. Counts were performed on 1.5 μm optical slices, with 3 slices measured and averaged for each polyp. For each slice, ectodermal and endodermal components were distinguished by eye and separate counts performed for the two tissues. Automated cell counts were performed using Volocity 6.0.1 software (PerkinElmer, Inc., Waltham, MA, USA). For each time point, average values were derived from counts on 5 to 10 individuals.

### Measurement of tentacle length

Tentacle morphology was determined by labeling filamentous actin in fixed polyps with BODIPY FL phallacidin (Molecular Probes, Eugene, OR, USA), and counterstaining nuclei with Hoechst 33342 (Invitrogen, Carlsbad, CA, USA). Confocal imaging was performed using a Zeiss LSM 710 microscope (Carl Zeiss Microimaging, Inc., Thornwood, NY, USA) with either a 20x/0.8 NA dry objective or a 40x/1.3 NA oil objective. Length of tentacles was determined using Volocity 6.0.1 software (PerkinElmer, Inc., Waltham, MA, USA), measuring from the base of the tentacle, at the level of the oral disc, to the tip. For animals where no tentacle buds could be distinguished, tentacle length was recorded as zero.

### Cell proliferation inhibitor treatments

Cell proliferation was blocked with two inhibitors, hydroxyurea (Sigma-Aldrich, St. Louis, MO, USA) and nocodazole (Sigma-Aldrich, St. Louis, MO, USA). Incubations with hydroxyurea were performed at a concentration of 20 mM. Incubations with nocodazole were performed at a concentration of 0.1 μM. For extended incubations, 1/3x seawater with inhibitor was exchanged with freshly diluted inhibitor every 12 hours. For pulse experiments, polyps were washed with 1/3x filtered seawater a minimum of four times after exposure to the inhibitor.

### Statistical analysis

Experimental data is presented as the mean ± standard error from at least three independent experiments. Data was analyzed with Student’s *t*-test, with the exception of comparisons between three time-series pulse treatments with hydroxyurea, in which case one-way ANOVA was performed. Differences with *p* value < 0.05 were considered significant.

## Abbreviations

dpf: Days post feeding; EdU: 5-ethynyl-2^′^-deoxyuridine; hpa: Hours post amputation; hpa2: Hours post secondary amputation; HU: Hydroxyurea; Noco: Nocodazole; wpf: Weeks post feeding.

## Competing interests

The authors declare that they have no competing interests.

## Authors’ contributions

YJP designed the study; performed experiments, microscopic imaging, and data to analyses; and drafted the manuscript. MQM participated in design of the study and assisted drafting the manuscript. Both authors read and approved the final manuscript.
